# Decision-Making Criteria of Odontectomy or Surgical Exposure in Impacted Maxillary Canine Based on Treatment Difficulty Index Modification

**DOI:** 10.1055/s-0041-1739447

**Published:** 2022-01-11

**Authors:** Olivia Jennifer Gunardi, Coen Pramono Danudiningrat, Andra Rizqiawan, Indra Mulyawan, Muhammad Subhan Amir, David Buntoro Kamadjaja, Ni Putu Mira Sumarta, Ganendra Anugraha, Reza Al Fessi, Liska Barus, Shigehiro Ono

**Affiliations:** 1 Department of Oral and Maxillofacial Surgery, Faculty of Dental Medicine, Universitas Airlangga, Surabaya, Indonesia; 2 Department of Oral and Maxillofacial Surgery, Graduate School and Institute of Biomedical and Health Sciences, Hiroshima University, Hiroshima, Japan

**Keywords:** treatment difficulty index, impacted maxillary canines, treatment prognosis, human and health

## Abstract

**Objective** Canine impaction is a difficult condition to treat, and it usually necessitates a combination of surgical exposure and orthodontic traction or surgical extraction. An accurate assessment of the maxillary canine's position can help determine the severity of the impaction, the difficulty of therapy, and the treatment's prognosis.

**Materials and Methods** A total of 55 impacted canines were studied and selected retrospectively. Difficulty indexes were used to measure the severity of impaction with pretreatment panoramic radiographs.

**Statistical Analysis** Pearson correlation was used to test the validity of the difficulty index modification score. Regression statistical analysis was used to evaluate any correlation between total scoring from each index with surgical treatment.

**Results** The validity test on the variable modification index score showed a valid value ( *p* = 0.000). According to both treatment difficulty and modification index, odontectomy group showed higher mean of total scoring than surgical exposure group. Treatment difficulty and modification index showed a significant correlation with surgical treatment ( *p* = 0.003 and *p* = 0.001).

**Conclusions** The higher the severity of canine impaction, the greater is the possibility of odontectomy than surgical exposure. Both indexes can consider to be used in determining surgical treatment planning.

## Introduction

The management of maxillary canines' impaction is very important to know, considering these teeth are the second most common teeth that have a tendency to impact after third molars, with a prevalence in the range of 1.1 to 13% of the population. 1 2 3 Surgery on these teeth is one of the most common surgical procedures in treatment planning, due to aesthetic and functional reasons. Because of the function of these canines, it is very important to be able to preserve the canines that are not fully erupted or predicted to be impacted, such as with surgical exposure and orthodontic traction. 4 5

Forced eruption of an impacted canines usually requires surgical and orthodontic intervention to allow the canines to reach the proper position in the dental arch without major damage to the other teeth. 6 But this method is not always possible. If the impacted canine cannot be preserved due to its location, an odontectomy may be considered. 7 8 9 Panoramic radiograph is the main routine investigation in cases of canine impaction, and is often combined with other radiological techniques to help diagnose and determine more accurate location of impacted canine. 10 11 Canine impaction require a very complex, multidisciplinary therapeutic management, considering its long treatment time, high cost, and many other factors that can affect the final treatment outcome. 6 12

Prognostic index developed by several researchers that estimates several important factors through the diagnostic process such as the prognosis of successful forced eruption treatment techniques, treatment duration, and level of difficulty. 2 13 14 An accurate and adequate evaluation of the position of the impacted maxillary canine is required to assist in decisions-making related to the severity of the position of the impacted teeth, the difficulty level of treatment, and the prognosis of the treatment. 2 6 14 But prognostic index has never been used to help determine the type of surgical treatment for the maxillary canines. In addition, the application of treatment difficulty index by Pitt et al 14 is too complicated, considering the weighting factor, so it is possible to be simplified by creating a new index as a modification of difficulty index to make it easier to obtain this index. Furthermore, this modified index is assessed to determine the validity of a decision, whether the impacted tooth requires surgical exposure or odontectomy. Based on the reasons above, this study was conducted with the aim of testing the validity of a newer modification difficulty index, which was originally made by Pitt et al, 14 and determining the correlation between the severity of the maxillary canine impacted teeth and the surgical treatments such as odontectomy and surgical exposure oral and maxillofacial surgery, which were evaluated using treatment and modification difficulty index.

## Materials and Methods

The study was conducted retrospectively based on the medical record data of patients who came to the Dental Hospital of Universitas Airlangga from 2014–2019. Ethical approval number 074/HRECC.FODM/III/2020 was obtained from the Ethics Committee of the Faculty of Dentistry, Universitas Airlangga.

Before the research began, it was preceded by creating a modification difficulty index. Treatment difficulty index for unerupted maxillary canine, which was explained by Pitt et al, 14 consist of the following nine factors: (1) age, (2) angulation to midline, (3) vertical position, (4) buccopalatal position, (5) horizontal position, (6) alignment of upper incisors, (7) space between upper lateral incisor and upper first premolar, (8) midline, and (9) rotation. This assessment was conducted by using pretreatment study models and radiographs of treated cases.

Counihan et al, 15 who made guidelines for the assessment of the impacted maxillary canine based on Pitt et al, mentioned four aspects of canine position which should be assessed along with carefully taking into account the age of the patient . The use of these prognostic factors in an index has been suggested to estimate treatment difficulty. These factors are: (1) overlap of incisor, (2) vertical height, (3) angulation, (4) position of apex. These criteria may aid decision-making regarding management of cases.

Diop Ba et al 16 conducted orthopantomographic analysis of the intraosseous position of the maxillary canines. In this study, the following four variables were used to characterize the spatial position of the right and left permanent maxillary canines: (1) angulation, (2) impaction depth, (3) mesiodistal position in relation to the ipsilateral incisor, and (4) mesiodistal position in relation to the ipsilateral premolar.

The modification index, which was used in this research, was created based on the above literature. The assessment was performed using panoramic radiograph as the main routine investigation in cases of canine impaction.

### Patient's Data Collection

The overall data of maxillary canine impacted patients who had undergone surgical treatment with odontectomy or surgical exposure were selected retrospectively based on inclusion and exclusion criteria. The inclusion criteria included: (1) patients with maxillary canines that have an impacted position or predicted to be impacted, (2) patients referred from orthodontist who had undergone an odontectomy or surgical exposure on impacted maxillary canine, (3) the patient must be at least 11 years old, (4) the patient's medical record is completed with preoperative panoramic radiograph and a complete clinical examination related to the maxillary canine impacted teeth, which has been discussed and approved by the supervising doctor in charge at that time. Meanwhile, the exclusion criteria were as follows: (1) patients with incomplete medical records and no preoperative panoramic radiographs, (2) patients with history of craniofacial abnormalities, congenital abnormalities, or syndromes, (3) impacted teeth with involvement of cysts, tumors, odontomas, or supernumerary teeth, and (4) patients with a history of previous orthodontic treatment.

All cases of impacted canines that met the inclusion criteria were then selected and studied using medical records and preoperative panoramic radiographs, 17 and then divided into 2 groups, that is, postodontectomy group and postsurgical exposure group. There are two kinds of difficulty indices used to measure the severity of the impacted canines, such as treatment difficulty index and modification difficulty index, which in this study was made into a relatively simpler index.

### Statistical Analysis

Data analysis was performed using SPSS software version 25; (Armonk, NY: IBM Corp). The collected data is presented descriptively in tabular form. The validity test on the modification difficulty index score was performed using the Pearson correlation test. Whether there was a correlation between treatment difficulty index score or modification difficulty index score and surgical treatment of odontectomy and surgical exposure was then determined with a regression test, with a significance level of *p* < 0.05. The value from area under the curve (AUC) is used to determine whether the treatment difficulty index and modification difficulty index score parameters are the right parameters in predicting surgical treatment, as well as to find the ideal cutoff value of each score in determining the surgical treatment, considering their sensitivity and specificity.

## Results

There was a total of 54 patients who were found complaining of maxillary canine impacted teeth and had undergone surgical treatment with either odontectomy or surgical exposure. There were 45 patients who met the inclusion criteria and were then included in this study. A total of 55 canines was then examined using preoperative panoramic radiographs, which were then divided into two groups, consisting of 23 cases of postodontectomy group and 32 cases of postsurgical exposure group. The results of the distribution data of the maxillary canine impacted teeth sample are shown in Table 1 and Table 2 .

**Table 1:** Demographic data using treatment difficulty index on maxillary canine impaction

Score	Variable	Surgical exposure group		Odontectomy group		Combined group	
		Number	Percentage	Number	Percentage	Number	Percentage
Age							
1	Less than 12 years	1	2.22%	0	0.00%	1	2.22%
2	12–15 years	2	4.44%	2	4.44%	4	8.89%
3	15–18 years	4	8.89%	2	4.44%	6	13.33%
4	Over 18 years	19	42.22%	15	33.33%	34	75.56%
Angulation to midline							
1	Less than 30 degrees	11	20.00%	3	5.45%	14	25.45%
2	30–45 degrees	5	9.09%	1	1.82%	6	10.91%
3	Over 45 degrees	16	29.09%	19	34.55%	35	63.64%
Vertical position							
1	Canine cusp tip at the level CEJ of the adjacent incisor	3	5.45%	1	1.82%	4	7.27%
2	Canine cusp tip at the middle of root the adjacent incisor	19	34.55%	5	9.09%	24	43.64%
3	Canine cusp tip within the apical third of root the adjacent incisor	10	18.18%	13	23.64%	23	41.82%
4	Canine cusp tip above the apical third of root the adjacent incisor	0	0.00%	4	7.27%	4	7.27%
Buccopalatal position							
1	Buccal	24	43.64%	13	23.64%	37	67.27%
1	Palatal	8	14.55%	10	18.18%	18	32.73%
Horizontal position							
1	Canine overlapping up to half the width of the lateral incisor	15	27.27%	5	9.09%	20	36.36%
2	Canine overlapping over half the width of the lateral incisor	2	3.64%	2	3.64%	4	7.27%
3	Canine completely overlapping the lateral incisor	3	5.45%	7	12.73%	10	18.18%
4	Canine overlapping up to half the width of the central incisor	12	21.82%	9	16.36%	21	38.18%
Alignment of upper incisors							
1	Incisors spaced	11	20.00%	12	21.82%	23	41.82%
2	Incisors well aligned	17	30.91%	9	16.36%	26	47.27%
3	Incisors crowded	4	7.27%	2	3.64%	6	10.91%
Canine space							
1	Over 7 mm	1	1.82%	2	3.64%	3	5.45%
2	4–7 mm	16	29.09%	4	7.27%	20	36.36%
3	2–4 mm	3	5.45%	4	7.27%	7	12.73%
4	0–2 mm	12	21.82%	13	23.64%	25	45.45%
Midline							
1	Midline coincident	14	25.45%	10	18.18%	24	43.64%
2	Midline displaced	18	32.73%	13	23.64%	31	56.36%
Rotation							
1	Rotation absent	29	52.73%	17	30.91%	46	83.64%
2	Rotation present	3	5.45%	6	10.91%	9	16.36%

Abbreviation: CEJ, cementoenamel junction.

**Table 2:** Demographic data using modification difficulty index on maxillary canine impaction

Score	Variable	Surgical exposure group		Odontectomy group		Combined group	
		Number	Percentage	Number	Percentage	Number	Percentage
Age							
1	Less than 12 years	1	2.22%	0	0.00%	1	2.22%
2	12–15 years	2	4.44%	2	4.44%	4	8.89%
3	15–18 years	4	8.89%	2	4.44%	6	13.33%
4	Over 18 years	19	42.22%	15	33.33%	34	75.56%
Angulation to midline							
1	Less than 30 degrees	11	20.00%	3	5.45%	14	25.45%
2	30–45 degrees	5	9.09%	1	1.82%	6	10.91%
3	Over 45 degrees	16	29.09%	19	34.55%	35	63.64%
Vertical position							
1	Canine cusp tip at the level of CEJ of the adjacent incisor	3	5.45%	1	1.82%	4	7.27%
2	Canine cusp tip at the middle of root the adjacent incisor	19	34.55%	5	9.09%	24	43.64%
3	Canine cusp tip within the apical third of root the adjacent incisor	10	18.18%	13	23.64%	23	41.82%
4	Canine cusp tip above the apical third of root the adjacent incisor	0	0.00%	4	7.27%	4	7.27%
Horizontal position							
1	Canine overlapping up to half the width of the lateral incisor	15	27.27%	5	9.09%	20	36.36%
2	Canine overlapping over half the width of the lateral incisor	2	3.64%	2	3.64%	4	7.27%
3	Canine completely overlapping the lateral incisor	3	5.45%	7	12.73%	10	18.18%
4	Canine overlapping up to half the width of the central incisor	12	21.82%	9	16.36%	21	38.18%
Position of apex							
1	Above canine position	11	20.00%	1	1.82%	12	21.82%
2	Above first premolar position	16	29.09%	8	14.55%	24	43.64%
3	Above second premolar position	5	9.09%	14	25.45%	19	34.55%

Abbreviation: CEJ, cementoenamel junction.

The validity test of modification difficulty index was then performed using Pearson correlation test by comparing the scores of each variable with the total score ( Table 3 ). This validity test showed the significance value of each variable modification difficulty index score of 0.000 ( *p* -value < 0.05), so it could be concluded that all of the score variables are valid.

**Table 3:** Validity test of modification difficulty index

Variable	*n*	Significance/ *p* -Value
Age	55	0.000
Angulation to midline	55	0.000
Vertical position	55	0.000
Horizontal position	55	0.000
Position of apex	55	0.000

**Fig. 1:**
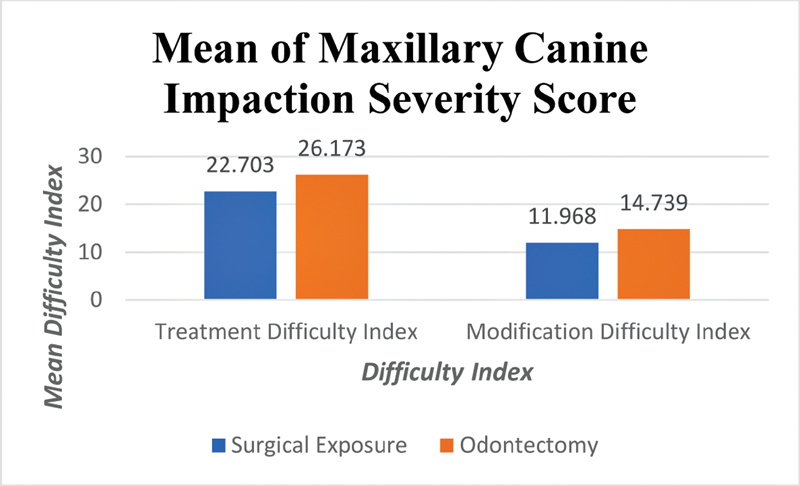
Column chart showed mean of maxillary canine impaction severity score.

The angulation of impacted teeth to the midline showed that the mean angulation in the odontectomy group (71.12 ° ± 40.50 °) was higher than the mean angulation in the surgical exposure group (36.94° ± 29.87°), with the mean angulation in the combined group was 51.23° ± 38.34°. The mean severity of maxillary canine impacted teeth using treatment difficulty index in the odontectomy group (26.173 ± 2.565) was higher than in the surgical exposure group (22.703 ± 4.321). The mean severity of maxillary canine impacted teeth using modification difficulty index in the odontectomy group (14.739 ± 1.763) was higher than in the surgical exposure group (11.968 ± 2.890). The severity of the maxillary canine impacted teeth according to the treatment difficulty index and modification difficulty index are shown in Fig. 1 .

**Fig. 2:**
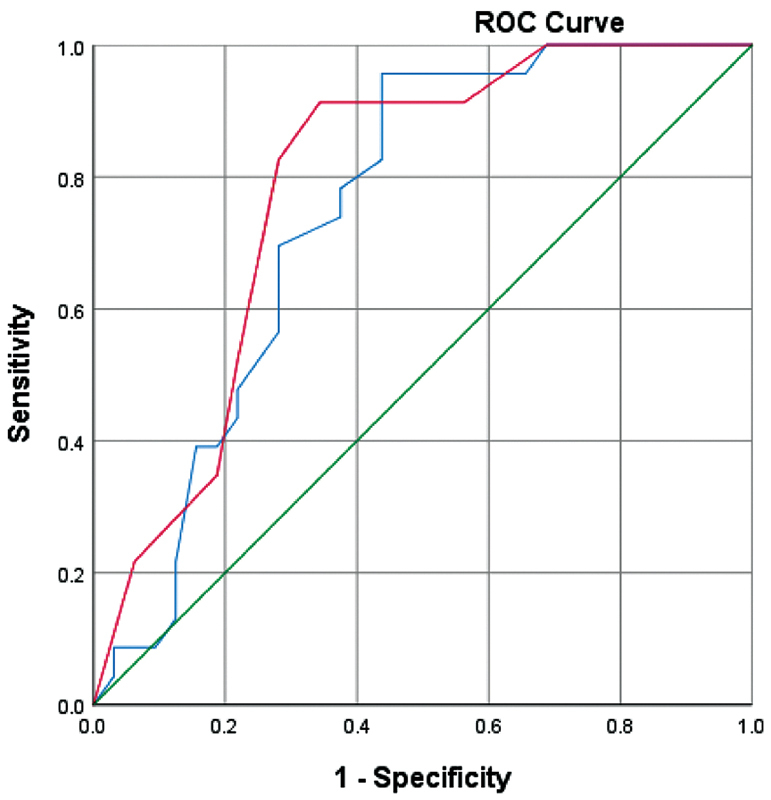
Receiver operator characteristic (ROC) curve for treatment difficulty index score (blue line), modification difficulty index score (red line), and reference line (green line). Diagonal segments are produced by ties.

The Hosmer and Lemeshow's goodness of fit test on the treatment difficulty index score 14 and modification difficulty index score showed a significance value of 0.095 and 0.109. There was no difference between the treatment difficulty index score and the modification difficulty index score ( *p* > 0.05) ( Table 4 ). The *p* -value of the logistic regression test showed whether there was relationship between the independent variable (the difficulty index score) on the dependent variable (surgical treatment of odontectomy or surgical exposure). The regression test results showed that the treatment difficulty index score and the modification difficulty index score were related to the surgical treatment of odontectomy or surgical exposure, with *p* -values of 0.003 and 0.001 ( *p* < 0.05) ( Table 5 ). The AUC results showed that the treatment difficulty index and the modification difficulty index score were quite good scoring parameters (0.7–0.8) in predicting surgical treatment ( Fig. 2 ). The AUC value of the modification difficulty index score (0.784) was slightly better than the AUC value of the treatment difficulty index score (0.747) ( Table 6 ).

**Table 4:** Results of Hosmer and Lemeshow's goodness of fit test

Variable	Significance/ *p* -Value
Treatment difficulty index score	0.095 a
Modification difficulty index score	0.109 a

a *p* -value < 0.05 showed significant differences.

**Table 5:** Logistic regression test results on difficulty index scores

Variable	Significance/ *p* -Value
Treatment difficulty index score	0.003 a
Modification difficulty index score	0.001 a

a *p* -value < 0.05 showed the influence or relationship between variables.

**Table 6:** AUC results of difficulty index score

**Variable**	**AUC**
Treatment difficulty index score	0.747
Modification difficulty index score	0.784

Abbreviation: AUC, area under the curve.

Then the cutoff value of the treatment difficulty index and modification difficulty index score was determined based on the coordinates of both index scores on the receiver operating characteristic (ROC) curve. The cutoff value of the treatment difficulty index and modification difficulty index score were used in determining the more needed surgical treatment (odontectomy or surgical exposure). The chosen cutoff value for the treatment difficulty index score was 25, and for the modification difficulty index score was 13.5. For the treatment difficulty index score, it was found that 69.6% of subjects had a chance to be true-positive, and 28.1% of subjects had a chance to be false-positive. While on the modification difficulty index score, it was found that 82.6% of subjects had the chance to be true-positive, and 28.1% of subjects had a chance to be false-positive.

Based on the cutoff value above, the modification difficulty index had a range of normal values with a score of 5 to 8, grade 1 with a score of 9 to 13.5 (more needed surgical exposure), and grade 2 with a score of 13.5 to 18 (more needed odontectomy).

## Discussion

The maxillary canines are the teeth that have a tendency to impact on the dental arch after the third molars, 15 18 19 so this is a challenge for orthodontists and oral and maxillofacial surgeons, especially in the treatment management and surgical approaches. Panoramic radiograph can help to predict maxillary canine impacted teeth, but cone-beam computed tomography (CBCT) can identify the location of maxillary canine impacted teeth precisely. 16 20

Another factor that affects the severity of impacted teeth based on this study is age. In this study, maxillary canine impacted teeth were seen more frequently in patients between 20 to 29 years with a percentage of 53.33%. According to Al-Abdallah et al, research growing older increases the chance of impacted teeth worsening in position, particularly when the angle of the tooth's long axis to the midline increases. In the elder age group, the angulation of affected canines was inferior. The findings of this study reveal that impacted teeth can migrate and pass through the midline over time, indicating the need of early identification and treatment planning. 21 The patient's age is an important factor for the forced eruptions during childhood and adolescence because impacted teeth can progressively develop into ankylosis, and orthodontic traction can become more difficult. 22

According to horizontal position, the severity of impacted teeth showed that the combined group and odontectomy group had the highest percentage of impacted teeth that overlapped up to half the roots of the central incisors, while the surgical exposure group had the highest percentage of impacted teeth that overlapped up to half the roots of the lateral incisors. This meant that the odontectomy group had a worse horizontal position than the surgical exposure group, and consequently had a worse prognosis. Only 64 percent of canines that looked to overlap with the lateral incisors of more than half of the roots (placed in sector 3 or more) could be positioned appropriately, compared with 91 percent of canines that appeared to overlap less than half of the roots (located in sector 3 or more). 15

The severity of impacted teeth based on angulation to the midline with a mean angulation of 51.23 °, in the surgical exposure group with a mean angulation of 36.94 °, and in the odontectomy group with a mean angulation of 71.12 °. This showed that the increase in angulation, as shown in the odontectomy group, had the greatest potential to increase the chance of surgical extraction of impacted teeth and thus had a worse prognosis. If angulation to the midline increases, the possibility of surgical extraction will also increase compared with forced eruptions. 23 The determination of prognosis by calculating the angulation of the long axis of the canine toward the midline orthopantomogram (OPG) that exceeds 31 ° will reduce the chance of spontaneous eruption after preventive treatment. 24

The highest percentage in the odontectomy group was 23.64 percent in the apical third of the lateral incisor roots, while the highest percentage in the surgical exposure group was 34.55 percent in the middle of the lateral incisor roots, according to the vertical position of the canine cusp tip. This meant that the odontectomy group's vertical position of the canine impacted teeth was worse than the surgical exposure group's, implying that the odontectomy group had a worse prognosis. The poorer the prognosis for orthodontic treatment, the more apical the crown position. When the cusp tip of the canine is in the cementoenamel junction (CEJ) of the adjacent incisor, the prognosis is good. 23 24

According to the findings of this study, the higher the difficulty index score of an impacted tooth location, the more difficult it is to align that tooth. 21 25 If the canines' prognosis was good in all areas, the primary canine could be extracted to allow the affected canine to spontaneously erupt. If the canine does not erupt within a year, orthodontic therapy including surgical exposure and alignment may be necessary. If the prognosis in these groups is mixed, definitive treatment with canine extraction can be conducted, depending on the total malocclusion and other relevant considerations such as patient age, crowding, and dentition condition. If one or more of the criteria is poor, or if disease is present, orthodontic treatment is required, and the primary canine should not be excised. Before settling on a definitive treatment in this circumstance, all considerations must be evaluated. 3 24 26

The fact that this is a retrospective study is one of the study's limitations. Furthermore, the number of samples was limited by inclusion criteria, and surgical exposure or odontectomy in patients was done by multiple different surgical operators. Given that the results of this study cannot be applied to other populations, more research is needed to validate or refute the findings, which should involve increasing the number of samples and conducting several investigations elsewhere.

## Conclusion

The severity of the maxillary impacted canine, as measured by the treatment difficulty index or the modification difficulty index, correlates with surgical treatment options such as odontectomy or surgical exposure. When comparing surgical exposure to odontectomy, the severity of the impaction determines the likelihood of odontectomy. Both indices demonstrate their impact and can be used to help determine surgical treatment options.
